# 

**Published:** 1907-02-16

**Authors:** 


### BOOKS RECEIVED.

Baillière, Tindall, and Cox.

"Aids to the Treatment of Diseases of Children." Third Edition. By J. McCaw, M.D.

"Cancer: its Treatment by Modern Methods. By Edmund Owen, Hon. LL.D.

H. K. Lewis.

"Anæsthetics: their Uses and Administration." By D. W. Buxton, M.D.

J. B. Lippincott Co.

"International Clinics." Vol. IV. Sixteenth Series.

Methuen and Co.

"The Evolution of Life." By H. Charlton Bastian, M.A., M.D.

Rebman, Ltd.

"The Treatment and Prophylaxis of Syphilis." By Alfred Fournier, translated by C. F. Marshall, M.D.

The Scientific Press, Ltd.

"The Care and Nursing of the Insane." Part I. By P. J. Baily, M.B.

"Skin Diseases, their Nursing and General Treatment." By G. Norman Meachen, M.D.

"Burdett's Hospitals and Charities," 1907.

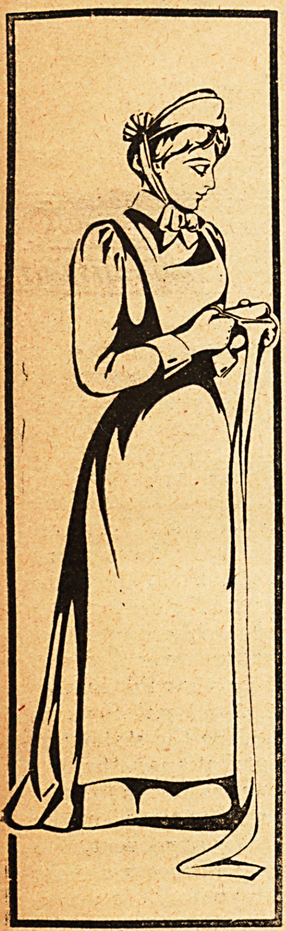

### HOW TO BECOME A NURSE

Many young women of the present day are, for one reason or another, attracted to Nursing as a career, but are at a loss to know how to enter the ranks of this important profession. **"How to Become a Nurse: The Nursing Profession, How and Where to Train,"** by Sir Henry Burdett, K.C.B. (price 2/4 post free), is the title of a most admirable work, which will be found to be a thoroughly reliable guide to this noble calling. It contains a list of the recognised Training Schools throughout the English-speaking world, with particulars respecting Hospital, Infirmary, District, and Military Nursing, and answers very completely all the enquiries of aspirants who would fain believe that they have a vocation for Nursing.

It is well, however, before entering upon a Probationership, to obtain some knowledge of the duties of a Nurse. These are very fully explained in **"Nursing: Hints to Probationers on Practical Work,"** by Mary Annesley Voysey (price 2/3 post free). From this book a very clear insight may be gained into the character of the training it is necessary to undergo, and of the qualities that are absolutely essential to success in this self-sacrificing profession.

Having decided to adopt Nursing as a profession, it would be advisable, whilst waiting for a vacancy in the Institution of your choice, or in the early stages of your probation, to acquire some knowledge of the elements of nursing, bandaging, and the structure and working of the human body. In this direction, **"Nursing: its Theory and Practice,"** by Dr. Percy Lewis (3/6 post free); **"A Practical Guide to Bandaging and Dressings,"** by Dr. Johnson Smith (2/- post free); **"Elementary Physiology for Nurses,"** by Dr. Marshall (2/- post free); **"Elementary Anatomy and Surgery for Nurses,"** by Wm. McAdam Eccles, M.S. (2/6 post free); and **"Elements of Anatomy and Physiology,"** by Dr. W. Bernard Secretan (2/3 post free), will be found most useful, and the information they convey is clear, concise, and accurate.

It is of course impossible to avoid the use of technical terms in such works as these, and it is therefore necessary that a good dictionary should be at hand for constant reference. **"The Nurse's Pronouncing Dictionary,"** by Honnor Morten (2/- post free), has been compiled especially for the use of Nurses, and contains the definition and pronunciation of most of the terms used in Medical and Nursing treatment. Its size also is a strong point in its favour, as it can very easily be carried in the apron pocket.

Another most useful work, and one it would be well to obtain, is **"Surgical Instruments and Appliances,"** by Harold Burrows, F.R.C.S. (1/8) post free). This book is of the utmost value to the Probationer, as by its assistance she will be able to readily identify the instruments and appliances used in various operations.

Many other important works on Nursing and kindred subjects will be found in the catalogue of The Scientific Press, Ltd., who give almost exclusive attention to the production of Nursing Textbooks; and the fact that their publications are in use in the foremost Training Schools is indisputable evidence of their value.

The Scientific Press, Ltd., 28–29 Southampton Street, Strand, London, W.C., will be pleased to send you, free of cost, on receipt of post-card, a copy of their latest Catalogue of Nursing Manuals, Charts, Case-Books, &c.

*NOW READY*

### SKIN DISEASES

THE NURSING AND GENERAL MANAGEMENT

BY

**G. NORMAN MEACHEN, M.D.,**

**B.S. Lond., M.R.C.P. Lond. and Edin., M.R.C.S. Eng.**

*SYNOPSIS OF CONTENTS.*

Anatomy and Physiology of the Normal Skin—Classification of Skin Diseases—The Different Lesions of the Skin—Principles of Diagnosis—Causes of Cutaneous Disorders—Factors in Diagnosis—The General System in Skin Complaints—The Urine in Skin Diseases—General Principles of Treatment—Lotions and Ointments—The Removal of Crusts—Use and Abuse of Soap—Antiseptics in Skin Diseases—The Influence of Diet—The Common Diseases of the Skin—Eczema—Psoriasis—Other Inflammatory Affections—Itching of the Skin an Important Symptom—Diseases, Parasitic and otherwise, that cause Pruritus—Vesicular and Bullous Diseases—Drug Eruptions—Ulcers of the Leg and their Special Treatment—Diseases of the Scalp and Face—Seborrhœa—Types of Baldness—Ringworm—Care of the Hair—Complexion Disorders—Lupus—Affections of the Sweat Glands—Skin Disease in Children—Special Features—Glossary and Brief Notes upon some of the Rarer Skin Diseases in General.

**Price 2s. 6d. net; 2s. 9d. post free.**

*Of all Booksellers.*

THE SCIENTIFIC PRESS, LTD.,
28 and 29 SOUTHAMPTON STREET, STRAND, LONDON, W.C.

